# Cryptic effects of biological invasions: Reduction of the aggressive behaviour of a native fish under the influence of an “invasive” biomolecule

**DOI:** 10.1371/journal.pone.0185620

**Published:** 2017-09-29

**Authors:** Laura Magliozzi, Frederico Almada, Joana Robalo, Ernesto Mollo, Gianluca Polese, Emanuel J. Gonçalves, Serena Felline, Antonio Terlizzi, Biagio D’Aniello

**Affiliations:** 1 Dipartimento di Scienze e Tecnologie Biologiche ed Ambientali, Universita`del Salento, Lecce, Italy; 2 Dipartimento di Biologia, Università degli Studi di Napoli “Federico II”, Napoli, Italy; 3 MARE – Marine and Environmental Sciences Centre, ISPA – Instituto Universitário, Lisbon, Portugal; 4 Istituto di Chimica Biomolecolare, Consiglio Nazionale delle Ricerche, Pozzuoli, Napoli, Italy; 5 Dipartimento di Scienze della Vita, Università degli Studi di Trieste, CoNISMa, Trieste, Italy; 6 Stazione Zoologica A. Dohrn, Napoli, Italy; University of Windsor, CANADA

## Abstract

The invasive green alga *Caulerpa cylindracea* has become an important component of the diet of the Mediterranean white seabream *Diplodus sargus*. As a consequence of this “exotic diet”, the algal bisindolic alkaloid caulerpin accumulates in the fish tissues. Although the compound shows structural similarity to endogenous indolamines that modulate animal behaviour, the potential impact of caulerpin on fish behaviour still remains unexplored. In this report, behavioural experiments both on groups and on single fish responding towards a mirror were performed under different doses of dietary caulerpin. Differences between treated and control groups for each behaviour and for the overall aggressive pattern during the different experimental phases showed that the aggressiveness of *D*. *sargus* decreased with the administration of caulerpin. These results call the attention to a still unexplored potential ability of bioactive metabolites from marine invasive species, to alter the behaviour on native species, with putative negative effects on patterns of fish growth and population dynamics.

## Introduction

The intentional or accidental introduction of alien species is emerging as one of the most dramatic impacts contributing to changes in biodiversity and ecosystem functioning accross the planet [1,2]. Such phenomena, called biological invasions, have deep impacts on the society with both ecological and economic costs [3]. A new and critical theme in invasion biology addresses how bioactive metabolites from invasive pests may impact marine biodiversity, and ecosystem functioning [4,5]. Moving in this research frame, recent studies suggested that secondary metabolites from the invasive green alga *Caulerpa cylindracea* (reported as *Caulerpa racemosa*) may produce complex indirect effects on the Mediterranean marine biodiversity [6–10]. In the last 20 years, *C*. *cylindracea* is showing a high invasive potential, with severe impact on benthic assemblages in the Mediterranean Sea [11–14]. *C*. *cylindracea* forms a net of ramified stolons growing on other algae and to covering the surface of invaded areas, decreasing species cover, number and diversity of the benthic macroalgal community [11,12,15,16]. Several species are observed to graze on *C*. *cylindracea*, and recently, Terlizzi and colleagues [7] have found that the alga is ingested by the white seabream *Diplodus sargus* (Linnaeus 1758) becoming, in some cases, the most important and abundant food item in its diet. As a consequence of the amount of *C*. *cylindracea* ingested, the white seabream tissues accumulate a bioactive algal secondary metabolite, namely the red pigment caulerpin [7–10]. Caulerpin is an alkaloid that shows a variety of important biological activities ([5] and the references therein), while its accumulation in fish tissues has been related to alteration of different cellular and physiological processes and also to modifications in lipid metabolism [9,10]. However, it is noteworthy that disruption of metabolic processes could profoundly influence normal fish behaviour, since metabolism is strongly associated with fish behavioural state [17]. Similarly, interference with several physiological systems from aquatic pollutants at levels well below those causing significant mortality, is known to cause alteration on complex behaviours of fish [18]. In addition, the structural similarities of caulerpin with endogenous amines showing critical affinity to neurological targets [19], strongly support the hypothesis of a possible impact of caulerpin on fish behaviour.

On this premise, potential behavioural changes in fish feeding on the invasive alga are expected. Changes in behavioural patterns in the white seabream [20] are likely to be important in crucial phases of feeding, reproduction and controlling shelters where they hide to escape predators. Interspecific and intraspecific interactions are strongly linked to ontogenetic patterns and life histories, which, in turn, depend on the performance of proper behaviours. Disruption of behaviours associated with predator avoidance, reproductive, and social interactions may impair a successful, adaptive life history strategy, posing serious risks to fish populations (e.g. [21]) and, through a mechanism of trophic cascade, to the functioning of the subtidal benthic community as a whole.

The impact of invasive species is, until now, mostly evaluated on the basis of direct competition and replacement of native species frequently disrupting entire communities ([22,3] and references therein). Native species that are actively feeding on invasive species are apparently coping or even thriving with those invasions. However, if changes in diet affect behaviour and only long-term effects on survival are expected this conclusion may prove to be misleading. Moreover, if a single bioactive natural product from invasive species can modify the behaviour of a key native species, we can reasonably expect that it will generate a succession of events that may produce delayed negative effects on the whole ecosystem.

The aim of this study was to test the effect of the administration of food enriched with purified caulerpin on the behaviour of captive juveniles of white seabream, in order to provide new insights on potential indirect effects of invasive species that would otherwise pass undetected and highlight the need for future studies to assess the impact of bioinvasions on wild fish populations.

## Matherials and methods

### Extraction of caulerpin in *C*. *cylindracea*

*C*. *cylindracea* was collected in Italy in the Gulf of Pozzuoli (40°48′N, 14°07′E) and stored at –20°C until chemical analyses were performed. The alga was exhaustively extracted with acetone at room temperature. The acetone extract was evaporated at reduced pressure and the residual water was extracted with diethyl ether. The diethyl ether extract was first fractionated on sephadex column (CHCl_3_/MeOH 1:1, as eluent) to give a fraction that was further purified by silica-gel column chromatography (gradient of light petroleum ether/Et_2_O, as eluent) to give pure caulerpin, identified by comparison of spectroscopic data with the literature [23]. Size-exclusion chromatography was achieved on Sephadex LH-20 column, whereas silica gel column chromatography was performed using Merck Kieselgel 60 powder. NMR experiments were recorded on a Bruker Avance-400 spectrometer using an inverse probe fitted with a gradient along the X-axis. The NMR spectra were acquired both in DMSO-d_6_, and in CDCl_3_.

No specific permission to collect the green alga at this site is required and the field studies did not involve endangered or protected species.

### Subjects and holding facilities

Juvenile white seabreams were collected in 2015 in the north-eastern Atlantic, central Portugal near Cascais (Parede: 38°40′N, 9°21′W). Fish were caught with hand nets in confined intertidal channels and large pools. All individuals from each sampling station were captured, stored in containers and randomly selected from those containers. Subsequently those individuals were transported in constantly aerated containers to the fish facility at ISPA-IU were they were measured, weighed and randomly assigned to test aquaria for behavioural observations. Since all captured fish were recently settled juveniles of the year there was a high uniformity of sizes along the behavioural experiments (supplementary material S1 Table). This study was performed in strict accordance with the recommendations of the Animal Care and Use Committee of ISPA-Instituto Universtário (ORBEA-ISPA; Permit Number: 01–2017) that specifically approved this study, and undertaken under the supervision of an accredited expert in laboratory animal science (following FELASA category C recommendations). Permission for capturing fish at the field site was granted by the Portuguese Environmental Agency (APA) and by local authorities (Cascais Environmental Agency—Cascais Ambiente—and Coast Guard—Capitania de Cascais). The field studies did not involve endangered or protected species.

At the end of the experiment fish were euthanized with an excessive dose of anaesthetics for posterior tissue analysis (MS222 tricaine methane sulphonate; Pharmaq, Norway).

In the laboratory, fish with a mean initial body weight of 1.82±0.1 g and a standard length of 3.99±0.1 cm were randomly housed in sea water aquariums (20×40×30 cm). Water quality was controlled for physicochemical parameters in order to keep them constant throughout the experiment. These parameters were: temperature, 20–22°C; dissolved oxygen, 7 mg L^-1^; pH 7–8; salinity 33–35 g L^-1^; NH_4_ and NO_2_ never exceeding 0.5 mg L^-1^.

### Food preparation

Test food was prepared by soaking 0.5 g of commercial pellet (stick for cichlids JBL, Joachim Böhme in Ludwigshafen) in 1.5 mL acetone, in which caulerpin was previously dissolved at the desired concentration and then evaporating the organic solvent under reduced pressure. The same procedure, without adding caulerpin, was performed for the control food.

A total of 84 individuals were observed, with 72 individuals being tested in group experiments and 12 individuals in mirror experiments.

### Experiment 1: Mirror test

During acclimation phase (10 days) fish were accustomed to the artificial food (stick for cichlids JBL, Joachim Böhme in Ludwigshafen) once a day in the morning. After acclimation, fish were randomly assigned to control (C), low dose mirror test (LD) and high dose mirror test (HD) groups. Four replicates of each of these conditions were performed using 12 tanks, each one containing 1 fish for an additional period of 15 days (treatment: 10 days; and post-treatment: 5 days). During the treatment phase, LD subjects were fed with 0.25 g of food enriched with caulerpin at natural estimated levels in *C*. *cylindracea* (0.1 mg g^-1^), while HD subjects were fed with a dose of caulerpin ten-fold higher (1.0 mg g^-1^). In the post-treatment phase fish were fed with 0.25 g of non-treated food. Residual food in the bottom of the aquaria was removed. All observation tanks had 3 opaque sides and a transparent front used for video recording. Each tank was equipped with an identical shelter made with half a tile. Fish were fed at 10:00 a.m. and videos of 5 min were recorded in two different sessions daily: at 9:00 a.m. (before feeding) and 11:30 a.m. (after feeding). Aggressive behavioural responses were triggered by introducing a mirror on each aquarium during the recording phase. Behavioural patterns were defined based on *D*. *sargus* ethograms available in the literature [24,20]. Duration and frequency of agonistic behaviours (threat, charging, tail beating, fight and bite) were recorded.

### Experiment 2: Group test

Preliminary experiments with randomly assigned groups of six juvenile *D*. *sargus* were performed in aquaria to determine the ideal experimental conditions. Size variation among fish within each group was low since all captured fish presented similar sizes along the experiment period (supplementary material S1 Table). Due to the high aggressive levels also reported by [20], the group experiments were designed for a shorter period compared to the mirror test (6 days, including treatment and post-treatment phases, of 3 days each). After an acclimation period of 3 days, fish were randomly assigned to control (C), low dose group test (LD) and high dose group test (HD) groups. Four replicates of each of these conditions were performed using 12 tanks without mirrors, each one containing 6 fish. The amount of food provided was increased to 0.5 g/day and residual food was removed. Video images were used to determine the duration and frequency of agonistic behaviours (threat, charging, tail beating, fight and bite) following each fish in the group separately in order to quantify individual sequences of behavioural patterns.

### Coding and analysis

Behavioural patterns were coded according to the descriptions provided in the literature for juvenile *D*. *sargus* [20,24]. Fish behaviour was analized with Solomon Coder beta^®^14.05.19 (ELTE TTK, Hungary). A second analysis of 25% of the video recorded was independently performed by another observer to verify the first analysis reliability. Agreement percentage was more than 95%.

Each aggressive behaviour was analysed individually to evaluate congruence of results.

A distance-based permutational multivariate analysis of variance (PERMANOVA; [25–26]) was performed to test hypotheses of differences in behavioural responses between treatments both in group and single experiments.

The design consisted of three orthogonal factors, namely Treatment (Tr; three levels, fixed), Session (Se; two levels, fixed) and During and Post-Treatment (DPT; two levels, fixed). Analyses were based on Euclidean distance measures on untrasformed data, and each test was performed by using 4999 random permutations of appropriate units [27–28]. Pair-wise comparisons were done when significant differences were detected (P <0.05).

As significant differences between Treatments were detected (see Results), a canonical analysis of principal coordinates (CAP [29–30]) was performed for the factor Treatment. Behavioural responses that might be responsible for any group differences seen in the CAP plot were investigated by calculating product-moment correlations of original variables (behavioural data) with canonical axes (e.g. [30]). These correlations of individual variables with the two canonical axes (r_1_ and r_2_) were then represented as lines in a projection biplot. All the analyses were performed using the computer program PRIMER version 6 [31], including the add-on package PERMANOVA+ [32].

## Results

Multivariate analyses provided evidence for statistically significant effect of caulerpin on behavioural responses of fish (Table 1) exposed for the longest period of time (10 days) to the metabolite. The non-significance of the Tr × Se × DPT interaction terms, indicated that differences among fish, both for duration and frequency, did not vary before and after meals across treatment and post-treatment phase (Table 1). The *post-hoc* comparison for the significant term Treatment showed a significant difference across all conditions (p < 0.05).

**Table 1:** Permutational multivariate analysis of variance. PERMANOVA analyzing differences in behavioral responses measured as duration and frequency among fish feeding food treated with caulerpin at low and high dose and controls. Differences are based on Euclidean dissimilarities of untransformed data. Each test was conducted using 4999 permutations of appropriate units. Analyses were performed with Type III (partial) sum of squares. Results of pairwise tests for the significant term treatment (Tr) are reported down in the table.

		Duration				Frequency		
Source	*df*	MS	Pseudo-F	P	*Df*	MS	Pseudo-F	P
Tr	2	1.5E5	20.2	***	2	27642	19.1	***
Se	1	52464	7.12	**	1	1552	1.1	Ns
DPT	1	3654.5	0.5	Ns	1	1800.9	1.2	Ns
Tr×Se	2	15307	2.1	Ns	2	782.2	0.5	Ns
Tr×DPT	2	1761.2	0.2	Ns	2	917.8	0.6	Ns
Se×DPT	1	10682	1.5	Ns	1	268.3	0.2	Ns
Tr×Se×DPT	2	1001.5	0.1	Ns	2	211.4	0.2	Ns
Res	284	7365.3			292	1448.5		
Total	295				303			
Pairwise tests for term Tr								
			C ≠LD≠ HD				C ≠LD≠ HD	

*ns* not significant.

* *p* < 0.05.

** *p* < 0.01.

*** *p* < 0.001.

Two canonical analysis of principal coordinates for the term Treatment was carried out, one for each level of the factor Session (before and after meals), both for duration and frequency recording.

In all of the cases, CAP achieved the highest allocation success (> 55%) using m = 5 principle coordinate (PCO) axes which explained 100% of variation in the original dissimilarity matrix.

**Fig 1:**
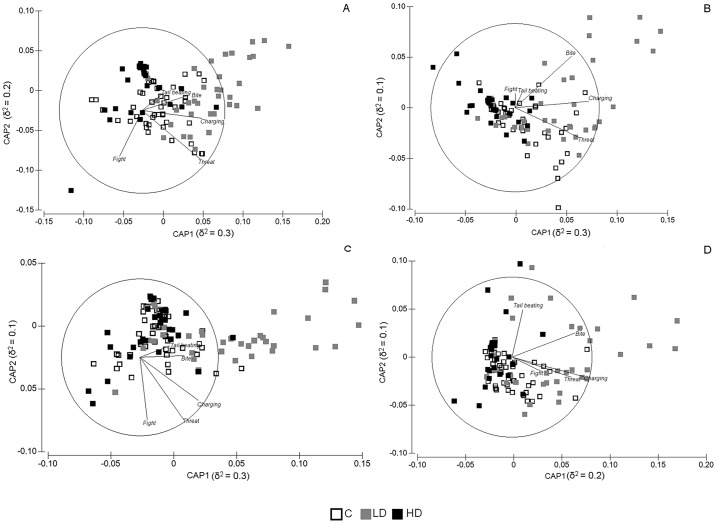
Canonical analysis of principal coordinates for the factor treatment. CAP ordination was obtained from the distance matrix among specimens on the basis of behavioural responses measured as Duration (A, B before and after feeding) and Frequency (C, D before and after feeding, respectively). Vectors represents the Pearson correlation of variables to axes (CAP 1 or CAP 2). The length of the vector is proportional to the strength of correlation.

Fig 1 summarizes the differences in the behavioural responses across the different levels of exposure to a caulerpin-based diet in the Mirror tests. Overall, there was a clear-cut separation between fish fed with high levels of caulerpin, which are clearly distinct along the first axis from fish of Low Dose group that, in turn, clustered together on the right-hand side of the graph.

Moreover, caulerpin at high dose seemed to reduce the high natural variability in the behavioural responses between specimens; a much higher scattering between specimens fed with low or null level of caulerpin with respect to fish treated with high dose of alkaloid was, indeed, observed (Fig 1). The behavioural responses most contributing to the differences observed between Treatments are Threat, Charging and Bite (Fig 1, Table 2). Specifically, the highest contribution to the observed differences was determined by Charging with a value of Pearson’s correlation with CAP1 axis equal to −0.7 and −0.9, before and after feeding, respectively, both for duration and frequency recording (Fig 1, Table 2). Finally, no significant differences were found between fish fed with different levels of caulerpin in group experiment.

**Table 2:** Correlation values with axes best discriminating between treatments in the principal coordinate space. Pearson correlation coefficients for all behavioural responses with CAP 1 or CAP 2 (indicated as vectors in Fig 1) as determined by canonical analysis of principal coordinates.

	Duration				Frequency			
	Before feeding		After feeding		Before feeding		After feeding	
	CAP1	CAP2	CAP1	CAP2	CAP1	CAP2	CAP1	CAP2
Threat	0.71	-0.59	0.77	0.36	-0.56	-0.80	-0.74	-0.25
Charging	0.72	-0.09	0.88	-0.08	-0.74	-0.55	-0.90	-0.25
Tail beating	0.23	0.19	0.08	-0.18	-0.38	0.15	-0.14	0.59
Fight	0.27	-0.55	0.00	-0.17	-0.09	-0.79	-0.39	-0.19
Bite	0.53	0.17	0.67	-0.61	-0.57	0.02	-0.78	0.30

## Discussion

This is the first study that attempts to analyze the effect of caulerpin from *C*. *cylindracea*, on aggressive behaviour of *D*. *sargus* under controlled conditions. Through mirror experiments, we showed clear-cut effects with duration and frequency of aggressive behaviour patterns responding inversely to the dose of caulerpin consumed, whereas no significant reduction in frequency and duration of aggressive behaviour towards other individuals was found in the group experiment. These contrasting results are not surprising and are attributable to the different duration of exposure to the metabolite in the two experiments. Group-level behaviour is more complex to analyze, in the sense that group effects are superimposed on individual variability. Unfortunately, due to the high aggressive levels reported in fish, we were forced to design the group experiments for a shorter period than the mirror test (three versus ten days of treatment), thus preventing from highlighting any clear effects of the metabolite on the behavioural pattern.

Mirror tests are potentially suitable for assessing aggressive behaviour since there is no evidence for self-recognition in fish [33]. Nevertheless the usefulness of mirror tests has been questioned because responses towards a reflected image may not elicit similar hormonal responses [34], brain activities [33] or even correlate with a response towards a real opponent [35]. However plenty of examples exist showing the suitability of mirror tests in fish allowing the suppression of uncontrolled sensory cues that may interfere with behavioural results [35–37]. In this experiment fishes were tested under identical conditions except for the dose of caulerpin administered with food. Results were congruent and highly significant in all individual fish tested and mirror experiments revealed that fish with higher doses of caulerpin reduced the frequency of displays and spent less time acting aggressively towards their image.

### Enlarge the scope—Indirect and elusive effects of bioinvasions

The results reported here are based on experiments performed on captive fish under controled conditions. Nevertheless, they raise ecosystem-level concerns related to the importance of the impacts over Mediterranean marine species. The inclusion of *C*. *cylindracea* in fish diet and, as a consequence, to its metabolites, is inducing changes in physiological processes of wild *D*. *sargus*, including alteration of lipid metabolism [9,10]. Our results provide an additional example of detrimental effects of bioinvasions that may remain underestimated or lead to misinterpretations since *D*. *sargus* is apparently thriving and actively feeding on this invasive alga [38,39,10].

Behavioural effects, such as the ones presented here, may have consequences on fish survival since variation in aggression among individuals can determine their relative position within social hierarchies, affecting life-history strategies, growth, reproductive success and survival [40–42]. Indeed, social interactions in fish, as well as in other vertebrates, are an important component of their social structure [43]. Social interactions, may have several direct impacts on individuals that compete for food and space [44,45,20] and may also affect the growth and the reproduction by neuroendocrine pathways [46]. Thus, changes in social interactions could have unpredictable effects on population dynamics.

While traits such as aggression influence dominance and ultimately survival, detailed knowledge of the physiological pathways underpinning these behavioural processes remain elusive [47]. Ultimate causation results reported here (e.g. behavioural changes due to the ingestion of caulerpin) should be complemented by proximate explanations on the mechanisms underlying these behavioural changes (e.g. which receptors in the nervous system are targeted by this metabolyte and which cascade effects are expected given those target cells). In this study, no evidence is presented on the physiological pathway of caulerpin and how it may influence behaviour. However, it is well known that this metabolite exerts an antinociceptive effect via pathways involving serotonin 5-HT_3_ receptors [48] and that serotonin (5-HT) activity is directly correlated to the frequency of agonistic behaviour in fish brain [43]. There was also a evidence of a correlation between serotoninergic activity and behaviour, suggesting a potential selective affinity of caulerpin for a neurological targets like the 5-HT subtypes with a key role in the modulation of aggressiveness [48].

Specific studies are needed to unravel the neurophysiological effects of caulerpin in fish. In addition, it is also important to verify whether a caulerpin enriched diet has similar effects on wild populations of *D*. *sargus*. If it does and if additional species show disruptive behavioural responses due to trophic shifts towards available food items in a changing environment, *D*. *sargus* may become a case study of cryptic effects of bioinvasions.

## Supporting information

S1 Table Means size and standard deviation of juvenile *D*. *sargus* in control, low dose and high dose groups. (DOCX)
