# Supplementary material for: Cryptic effects of biological invasions: Reduction of the aggressive behaviour of a native fish under the influence of an “invasive” biomolecule
Source: PLoS One. 2017 Sep 29;12(9):e0185620. doi: 10.1371/journal.pone.0185620 (PMC5621679; doi:10.1371/journal.pone.0185620)
Supplement: S1 Table — (DOCX) [file pone.0185620.s001.docx]

| **Mirror Test** | | | | |
| --- | --- | --- | --- | --- |
|  | Total Lenght (cm) | Standard Lenght (cm) | Height  (cm) | Weight  (g) |
| \| **Control** \| \| --- \| | 5.44 ± 0.39 | 4.28 ± 0.27 | 1.58 ± 0.23 | 2.17 ± 0.45 |
| \| **Low Dose** \| \| --- \| | 5.58 ± 0.40 | 4.41 ± 0.28 | 1.73 ± 0.16 | 2.18 ± 0.42 |
| **High Dose** | 5.48 ± 0.35 | 4.36 ± 0.39 | 1.74 ± 0.19 | 2.25 ± 0.48 |

| **Group Test** | | | | |
| --- | --- | --- | --- | --- |
|  | Total Lenght (cm) | Standard Lenght (cm) | Height  (cm) | Weight  (g) |
| \| **Control** \| \| --- \| | 4.67 ± 0.71 | 3.67 ± 0.60 | 1.39 ± 0.25 | 1.42 ± 0.60 |
| \| **Low Dose** \| \| --- \| | 5.20 ± 0.66 | 4.16 ± 0.57 | 1.60 ± 0.22 | 1.98 ± 0.84 |
| **High Dose** | 4.94 ± 0.89 | 3.93 ± 0.71 | 1.56 ± 0.29 | 1.79 ± 0.93 |

**S1 Table.** Means size and Standard Deviation of juvenile *D.sargus* in Control, Low Dose and High Dose groups.
